# Contribution of proprioceptors in the mesencephalic trigeminal nucleus and their surrounding astrocytes to acidic saline-induced chronic jaw muscle pain in rodents

**DOI:** 10.3389/fncel.2026.1800565

**Published:** 2026-04-01

**Authors:** Masaharu Yamada, Dorly Verdier, Arlette Kolta

**Affiliations:** 1 Département de Neurosciences, Faculté de Médecine, Université de Montréal, Montréal, QC, Canada; 2 Centre Interdisciplinaire de Recherche sur le Cerveau et l'Apprentissage (CIRCA), Montréal, QC, Canada; 3 Faculté de Médecine Dentaire, Université de Montréal, Montréal, QC, Canada

**Keywords:** acidic saline-induced pain, astrocyte, chronic muscle pain, S100β, trigeminal mesencephalic nucleus

## Abstract

**Introduction:**

Chronic jaw muscle pain is a common clinical condition whose etiology remains ill-defined. Using acidic saline injections into the masseter muscle to mimic it, we examined the hypothesis that hyperexcitability of jaw closing muscles spindle afferents (MSA) that have previously been observed in this model result from neuron glia interactions in the trigeminal mesencephalic nucleus (NVmes) lead to activation of nociceptive pathways.

**Methods:**

This was assessed using whole-cell patch-clamp recordings from NVmes neurons combined to pharmacological and astrocytic optogenetic stimulations and immunohistochemistry against cFos in the ventrolateral pole of the subnucleus interpolaris/caudalis transition region (vl-Vi/Vc), and GFAP in vl-Vi/Vc and NVmes regions in rats and mice

**Results:**

Acidic saline injection into the masseter muscle led to increases in: (1) cFos expression in vl-Vi/Vc at 9 days after the injection, (2) reactivity of astrocytes in NVmes, and (3) Excitability of NVmes neurons that manifested spontaneously or in response to astrocytic stimulation. This increased activity is thought to result from the release of the astrocytic Ca^2+^-binding protein S100β, since it was not observed in S100β knock-out mice, which also did not show increased expression of cFos in vl-Vi/Vc, despite showing increased reactivity of NVmes astrocytes.

**Discussion:**

These findings suggest that acidic saline injection into the masseter muscles induced long-term activation of astrocytes in the NVmes and promoted ectopic firing of NVmes neurons via astrocyte-released S100β, and subsequent activation of nociceptive pathways.

## 1 Introduction

Chronic muscle pain, called myofascial pain syndrome, not only causes suffering to patients but is also recognized for its association with high unemployment rates among these patients (Landmark et al., 2013) and the substantial healthcare burden they impose (Gaskin and Richard, 2012). Consequently, it has been highlighted that addressing chronic muscle pain should be a high priority (Breivik et al., 2013), yet its causes and etiology are still poorly understood.

To mimic the myofascial pain and delayed-onset muscle soreness (DOMS) following eccentric or intensive exercise, Sluka et al. (2001) developed a model of muscle pain induced by injecting acidic saline into the gastrocnemius muscle of rats. This model is considered useful for understanding myofascial pain syndrome because pain persists even after the muscle tissue pH returned to normal and because this type of soreness closely resembles the reports of patients suffering from chronic jaw muscle pain, low back pain, and other muscle pain (Hood et al., 1988).

Several studies suggest that acid-sensing ion channels (ASICs) play an important role in DOMS (Fujii et al., 2008; Gazerani and Cairns, 2018; Lee et al., 2025). These are expressed in both small-diameter PA and MSA in muscles (Lin et al., 2016; Gazerani and Cairns, 2018; Lee et al., 2025), but interestingly, acid-induced hyperalgesia persisted in the conditional knockout of ASIC3 in small-diameter PAs mice, but not in the conditional knockout of ASIC3 in MSA (Lee et al., 2025), indicating the involvement of proprioceptors in acid-induced chronic myofascial pain rather than nociceptors. Other lines of evidence suggesting that these large-diameter PAs, whose activity is normally not related to pain, may also contribute to chronic pathological pain include the facts that: (1) mechanical, but not thermal, hypersensitivity develops normally after nerve injury in mice in which transmission from nociceptors is specifically prevented genetically (conditional VGluT2 knock-outs) (Scherrer et al., 2010), (2) specific activation or large diameter PAs with vibration or light mechanical stimuli produces pain after DOMS or peripheral tissue or nerve injury (Zhu and Henry, 2012; Colloca et al., 2017), and (3) In humans, as in several animal neuropathic pain models, pain onset coincides with appearance of ectopic firing in these afferents (Chul Han et al., 2000; Liu et al., 2000; Khan et al., 2002; Tal et al., 2006). However, besides the observation that specific stimulation of MSA with vibration at 80 Hz increased muscle pain after DOMS in the triceps surae muscle of humans (Weerakkody et al., 2001) it was unknown whether excitability changes leading to ectopic firing occur only after nerve lesion or could also appear after milder damage such as those producing muscle soreness and pain.

Chronic jaw muscle pain is one type of chronic myofascial pain syndrome. Acute jaw muscle pain, often described as inflammation or injury of jaw muscles, is experienced by 10% of the population (LeResche, 1997) and becomes chronic in some cases where it persists after extinction of inflammation. Lund et al. (2010) adapted Sluka’s model to the jaw closing muscles to further investigate mechanisms potentially underlying chronic jaw muscle pain and found that injection of acidic saline into the masseters (jaw closing muscles) of rats induced mechanical hypersensitivity for up to 5 weeks afterwards. Interestingly, this change in mechanical sensitivity was paralleled by changes in excitability of neurons in the trigeminal mesencephalic nucleus (NVmes) (Lund et al., 2010), which contains the somata of jaw closing MSA. They postulated that the ectopic firing resulting from this hyperexcitability could travel antidromically in the peripheral branches of the MSA and induce glutamate release in the spindle capsule. The released glutamate could then activate nociceptors’ free endings found in the vicinity of these release sites. They further provided anatomical evidence that nociceptive endings carrying glutamatergic metabotropic receptors (mGluR5) can be found closely opposed to peripheral endings of MSA containing the glutamate vesicular transporter VGlut1; thereby supporting the hypothesis.

Firing of NVmes neurons always emerges from subthreshold membrane oscillations (SMOs) (Verdier et al., 2004; Wu et al., 2005; Enomoto et al., 2007; Gaudel et al., 2025) which rely on a sodium persistent current (I_NaP_) (Wu et al., 2001, 2005; Enomoto et al., 2007; Gaudel et al., 2025) whose amplitude increase with reduction of extracellular Ca^2+^ concentration ([Ca^2+^]_e_) (Gaudel et al., 2025). Astrocytes are known to release the calcium-binding protein S100β, which by decreasing [Ca^2+^]_e_ enhances I_NaP_-mediated changes of excitability of nearby neurons (Morquette et al., 2015; Ryczko et al., 2021). Gaudel et al. (2025) applied these findings to NVmes in mice and assessed whether astrocytes and their S100β can produce such a decrease in [Ca^2+^]_e_ in NVmes (Gaudel et al., 2025). They showed that local applications of S100β or BAPTA (a Ca ^2+^ chelator that decreases [Ca^2+^]_e_) near the stem axon, where the channel mediating I_NaP_ is highly concentrated, caused ectopic firing. These effects were reproduced by optogenetic stimulation of astrocytes near the stem axon and were mediated by the release of S100β and I_NaP_ activation.

Since many studies reported activation of astrocytes (as detected by markers of reactivity) (Okada-Ogawa et al., 2009; Borghi et al., 2023; Yang et al., 2026) in conditions of pathological pain, we hypothesized that the hyperexcitability and ectopic firing of NVmes neurons associated with the acid-induced mechanical hypersensitivity result from astrocytic reactivity and release of S100β.

Here, using immunohistochemistry against the activity marker cFos (cellular oncogene fos) and astrocytic reactivity marker GFAP (glial fibrillary acidic protein) and electrophysiological recordings of NVmes neurons, we first validated that findings previously obtained in rats could be reproduced in mice to enable use of transgenic mice lines engineered to allow manipulation of astrocytes. Our results confirm findings previously reported by Lund et al. (2010) in rats and suggest that S100β may be involved in the observed effects (Lund et al., 2010).

## 2 Methods

### 2.1 Animals

All experiments were conducted according to the Canadian Institutes of Health Research rules and were approved by the Animal Care and Use Committee of Université de Montréal.

A total of 12 rats and 60 mice were used, including 30 wild type (WT) mice (C57BL/6 J, Stock 000664, JAX), 21 mice expressing the channelrhodopsin 2 (ChR2) under the control of the GFAP promoter (GFAP-ChR2-EYFP mice) and 9 S100β null mice (S100β KO mice) (Table 1). GFAP-ChR2-EYFP mice were produced by crossing GFAP-Cre (B6. Cg-Tg(Gfap-cre)73.12Mvs/J, stock 12,886, JAX;105) and ChR2-lox mice [B6. Cg-Gt(ROSA)26Sortm32(CAG COP4∗H134R/EYFP)Hze/J, stock 24,109, JAX;106]. The S100β null mice were obtained by crossing heterozygous B6Brd; B6N-Tyrc-Brd S100btm1a(EUCOMM)Wtsi/WtsiCnbc mice (Wellcome Trust Sanger Institute) and selecting the homozygous offspring. Experiments were performed using both sexes, and sex was not factored into the analysis, because a clinical study suggests that there is no difference between sexes in chronicisation of temporomandibular disorders (Nguyen et al., 2019).

**Table 1:** Animals and treatment.

Animal group	Treatment		Experiment	
	Neutral saline (CTL)	Acidic saline (PAIN)	Immunohistochemistry	Electrophysiology
Rats (*N =* 12)	6	6	12	0
WT mice (*N =* 30)	15	15	7	23
GFAP-ChR2-EYFP mice (*N =* 21)	8	13	0	21
S100β KO mice (*N =* 9)	4	5	6	3

ChR2, channelrhodopsin 2; EYFP, enhanced yellow fluorescent protein; GFAP, glial fibrillary acidic protein; KO, knock-out; N, number of animals; WT, wild type.

Mice and rats were separated into 2 groups randomly (Table 1). One group (CTL, control group) was injected with 20 μL saline (pH 7.4) into both sides of the masseter muscle. The other group (PAIN, pain group) was injected with 20 μL acidic saline (pH 4). Following the protocol established by Lund et al. (2010), the injections were repeated 2 days later, that is, at postnatal day 8 and 10 in rats (12–22 g), postnatal days 7 and 9 in mice (around 4–5 g) for electrophysiological experiments, between postnatal 70 and 100 days in mice (17–25 g) for immunohistology experiments under isoflurane anesthesia. A blind person conducted injections throughout all experiments.

### 2.2 Immunohistochemistry

Rats were perfused 5 and 9 days after the second injection. Mice were subjected to the same procedure at 9 days post-injection. Stimulation of masseter muscles on both sides was conducted, 20 times using the 15 g Von-Frey filament by a blind experimenter under light urethane anesthesia, 90 min before the perfusion. The rats and mice were then deeply anesthetized again with urethane and perfused intracardially with saline, followed by 4% paraformaldehyde in phosphate-buffered saline (PBS) on the respective day. The brains were quickly removed and post-fixed overnight with 4% paraformaldehyde at 4 °C. For brain sectioning, the brains were first separated between the NVmes and the trigeminal spinal nucleus levels. For sectioning of the trigeminal spinal nucleus in rats at 9 days after the 2nd injection, the brains were submerged in 20% sucrose PBS overnight after post-fixation, and sectioned coronally at 50 μm using a cryostat (Leica CM3050 S, Leica Biosystems, Nussloch, Germany). For the other parts of the brains in rats and mice, the brains were embedded in 3% agarose just before sectioning and were sectioned coronally at 80 and100 μm at the NVmes level of mice and rats, respectively and 50 μm at the trigeminal spinal nucleus level of both rats and mice using a vibratome (Vibratome 1,000, Technical Products International Inc., St. Louis, MO, USA). For immunostaining, all steps were carried out at room temperature unless specified otherwise. Tissue sections were rinsed with PBS containing 0.5% Triton-X (Fisher Scientific, Waltham, MA, USA) and removed from embedded agarose or 20% sucrose-PBS, followed by a 120-min incubation in a blocking solution containing 5% normal donkey serum and 0.5% Triton X-100 in PBS. This solution was also utilized for all antibody dilutions. Sections were then incubated overnight at 4 °C in primary antibodies (see Table 2). Then, secondary antibodies were applied for 2 h (see Table 2). All sections were mounted on glass slides (Fisherbrand Superfrost Plus, Fisher Scientific, Waltham, MA, USA). In the negative controls, the primary antibodies were omitted, and in each case, there was no detectable labelling. Within a few days, the sections were observed and captured under the Colibri 7 solid-state LED light source (Carl Zeiss, Oberkochen, Germany).

**Table 2:** Sources of antibodies.

Antibodies	Source	Identifier
GuineaPig anti-Parvalbumin	SynapticSystems	Cat: #195308, RRID: 1–9 Cat: #195308, RRID: 1–10
Chicken anti-GFAP	Abcam	Cat: #AB4674, RRID: 1118849–3 Cat: #AB4674, RRID: 1012209–1
Mouse anti-NeuN	Millipore	Cat: #MAB377, RRID: 3832727
Rabbit anti-cFos	Abcam	Cat: #AB190289, RRID: GR3393951-1
Donkey anti-Chicken Alexa Fluor 488	Jackson ImmunoResearch	Cat: #703–545-155, RRID: 170532
Donkey anti-Rabbit Alexa Fluor 488	Jackson ImmunoResearch	Cat: #711–545-152, RRID: 172897
Donkey anti-Mouse Alexa Fluor 594	Jackson ImmunoResearch	Cat: #715–585-151, RRID: 144883
Donkey anti-GuineaPig Alexa Fluor 594	Jackson ImmunoResearch	Cat: #706–585-148, RRID: 170762
Donkey anti-GuineaPig Alexa Fluor 647	Jackson ImmunoResearch	Cat: #706–605-148, RRID: 158548
Donkey anti-chicken Alexa Fluor 594	Jackson ImmunoResearch	Cat: #703–585-155, RRID: 111794

### 2.3 GFAP image analysis

**Figure 1:**
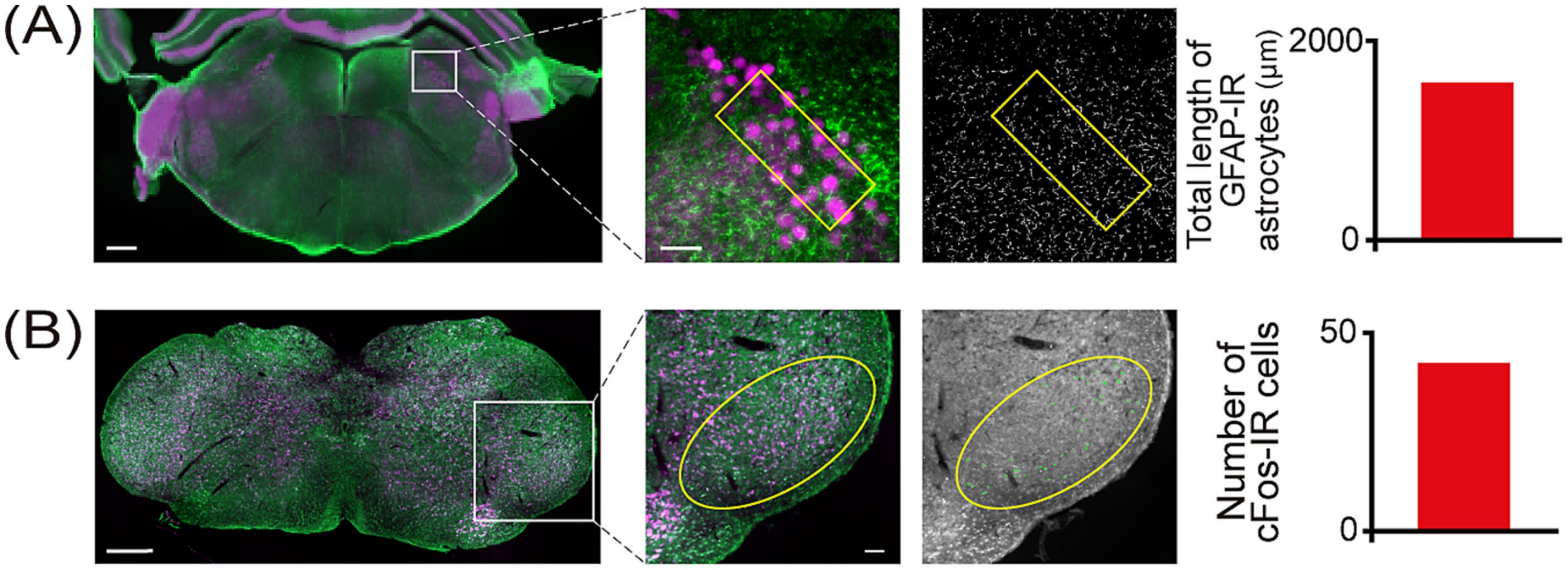
Histological analysis. **(A)** Left: Photomicrograph of a representative brainstem section at low magnification showing the NVmes nucleus (white square). The scale bar represents 500 μm. Middle: GFAP-IR astrocytes (green) in the NVmes. NVmes neurons are immunoreactive to parvalbumin (magenta). The scale bar represents 100 μm. Right: A skeletonized version of the middle image processed using the Fiji plugin. The yellow box indicates the ROI. The graph on the right shows the total length of GFAP-IR astrocyte processes within the ROI. **(B)** Left: Photomicrograph of a representative brainstem section at low magnification showing the vl-Vi/Vc (in the white square). The scale bar represents 500 μm. Middle: Examples of cFos-IR cells in the vl-Vi/Vc (yellow ellipse); (magenta: NeuN; green: cFos). The scale bar represents 100 μm. Right: Auto-detected cFos-IR cells processed using the Zen software. The yellow ellipse indicates the vl-Vi/Vc area (ROI). The graph on the right shows the total number of cFos-IR cells within the ROI. cFos-IR, cellular oncogene fos-immunoreactive; GFAP-IR, glial fibrillary acidic protein-immunoreactive; NeuN, neuronal nuclei; NVmes, trigeminal mesencephalic nucleus; ROI, region of interest; vl-Vi/Vc, ventrolateral pole of the subnucleus interpolaris/caudalis transition.

GFAP which is often used as a marker of astrocyte activation (Okada-Ogawa et al., 2009; Borghi et al., 2023; Fonseca-Rodrigues et al., 2026; Yang et al., 2026) was first measured in the caudal part of NVmes containing the highest densities of proprioceptors’ somata. These can be easily identified by parvalbumin immunostaining (Gaudel et al., 2025). Images of the NVmes were acquired with a 20x objective. Two sections were used in rats, while only one at a comparable level was used in mice (due to the smaller size of their NVmes). In each section, measurements were made within a Region of Interest (ROI; Figure 1A) of 150 μm x 400 μm in rats, and 60 μm x 250 μm in mice (again due to the smaller size of their NVmes), on each side of the section. The ROIs were positioned by a blind experimenter and with great care to avoid inclusion of the Locus coeruleus and Parabrachial nucleus adjacent to NVmes. Data from the left and right sides were analyzed as independent samples, and in the cases having two sections per animal (rats), the data were averaged for each side. The astrocytes that were running along blood vessels were excluded from analysis.

GFAP measurements were also made in the ventrolateral pole of the subnucleus interpolaris/caudalis transition region (vl-Vi/Vc) which is known to play an important role in deep orofacial pain (Wang et al., 2006; Nakatani et al., 2018), vl-Vi/Vc sections were taken at the level of the obex. Images were acquired at 40x magnification for rats and 64x for mice. As vl-Vi/Vc is a large region and all acquired images were considered to belong to vl-Vi/Vc, the entire acquired image (312 μm × 312 μm for rats, and 198 μm × 198 μm for mice) area was analyzed as the ROI.

Skeletonization of the GFAP staining was performed using the Fiji distribution of ImageJ (NIH, USA) according to the method by Marques et al. (2023). Briefly, the images were converted to binary images by applying a threshold calculated from the histogram of the image fluorescence intensity using the MaxEntropy algorithm, after image processing by the method of Marques et al. (2023). Subsequently, the Skeletonize plugin of ImageJ was utilized, followed by the elimination of GFAP-immunoreactive (GFAP-IR) astrocyte segments shorter than 5 μm. Finally, the total length of GFAP-IR astrocytes residing within the ROI was calculated (Figure 1A).

### 2.4 cFos image analysis

Images of the vl-Vi/Vc were acquired with a 20x objective. cFos-immunoreactive (cFos-IR) cell counts in the vl-Vi/Vc region were automatically quantified using ZEN software (Carl Zeiss, Oberkochen, Germany) (Figure 1B). The automatically extracted cells were confirmed, and any label clearly not regarded as cFos-IR cells were excluded by a blind person.

### 2.5 Brainstem slice preparation

Using a VT1000S vibratome (Leica), coronal brainstem slices (350 μm) were prepared from mice aged 13–36 days. The control and pain groups were age-matched, more or less 2 days, and most often were from the same litter. Following anesthesia by isoflurane (Pharmaceutical Partners of Canada Inc., Richmond Hill, ON, Canada) inhalation, the mice were decapitated and their brain were rapidly extracted and sectioned in an ice-cold modified artificial cerebrospinal fluid (CSF, in mM: 3 KCl, 1.25 KH_2_PO_4_, 4 MgSO_4_, 26 NaHCO_3_, 10 Dextrose, 0.2 CaCl_2_, 219 Sucrose, pH 7.3–7.4, 300–320 mOsmol/kg) saturated with a mix of 95% O_2_ and 5% CO_2_. After sectioning, the slices were kept at room temperature in a holding chamber filled with artificial CSF (in mM: 124 NaCl, 3 KCl, 1.25 KH_2_PO_4_, 1.3 MgSO_4_, 26 NaHCO_3_, 10 Dextrose, and 1.6 CaCl_2_, pH 7.3–7.4, 294–300 mOsmol/kg) bubbled with 95% O_2_ and 5% CO_2_.

### 2.6 Electrophysiology and analysis

The experimenter was blinded to the experimental groups throughout all electrophysiological recordings. For recordings, one slice was transferred to a submerged chamber continually perfused with artificial CSF bubbled with 95% O_2_ and 5% CO_2_. Patch microelectrodes (resistance 6–10 MΩ) were pulled from borosilicate glass capillaries (1.5 mm outside diameter, 1.12 mm inside diameter, World Precision Instruments) using a P-97 puller (Sutter Instruments). For neuronal recordings, pipettes were filled with an internal solution containing (in mM): 140 K-gluconate, 5 NaCl, 2 MgCl_2_, 10 HEPES, 0.5 EGTA, 2 Tris ATP salt, 0.4 Tris GTP salt, pH 7.2–7.3, 280-300mOsmol/kg. Alexa Fluor 488 or 594 was added to the internal solution to visualize neuronal and axonal morphologies during the experiment. We used an Olympus Fluoview FV 1000 confocal microscope equipped with a 40x (N. A. 0.80) water immersion objective for imaging. All recordings were performed using a Multiclamp 700A amplifier, Digidata 1322A interface coupled to a computer equipped with pClamp 8 or 11 software (Molecular Devices, San Jose, CA). The pipette resistance and capacitance were compensated electronically. Neurons were discarded when action potentials did not overshoot 0 mV or when the resting membrane potential was depolarized (> − 45 mV). Standard scripts in Clampfit were used for analysis. The input resistance was determined as the slope of the linear part of the current–voltage (I-V) curve.

### 2.7 Optogenetic stimulation

Two lasers (440 and 488 nm) were used simultaneously in the SIM lightpath of an FV1000 microscope (Olympus) for optogenetic stimulation of the astrocytes in GFAP-ChR2-EYFP mice. The SIM scanner was used in normal scanning mode to photoactivate manually delineated small areas surrounding the recorded neuron. Optogenetic stimulations were applied using 30 s pulses (10–20% laser power/8.6–14.9 mW for laser 440 nm/8.7–15.9 mW for laser 488 nm).

### 2.8 Drug application

1,2-bis(o-aminophenoxy)ethane-N, N, N0, N0-tetraacetic acid tetrasodium salt [BAPTA, 5 mM; Sigma-Aldrich (Oakville, Ontario, Canada)] diluted in artificial CSF was locally applied with glass micropipettes (tip diameter around 2 μm) with 2–20 psi pressure pulses of variable duration (1–30 s, Picospritzer III, Parker Instrumentation, Fairfield NJ USA).

### 2.9 Statistics

Statistical analyses were performed using the commercial software SPSS (IBM, NY, USA). Normality was assessed using the Shapiro–Wilk test with a significance level of *α* = 0.05. For non-normally distributed data, the Mann–Whitney U test was performed. When normality was confirmed, statistical significance was determined using either Student’s *t*-test (for equal variances) or Welch’s *t*-test (for unequal variances), depending on the results of Levene’s test for homogeneity of variance (*α* = 0.05). For categorical data, Fisher’s exact test was used when expected frequencies were <5. Otherwise, the *χ*^2^ test was applied. In all immunohistochemical experiments, two or three animals were analyzed per group for each parameter, and data were analyzed separately for the left and right sides to minimize animal use. In all electrophysiological experiments, to minimize animal use as well, we confirmed that no marked inter-animal differences were observed and therefore recorded multiple NVmes neurons (n: number of cells) from a single mouse (N: number of animals), finally pooling them as samples. All data are presented as mean ± standard error to the mean (SEM) and as proportions (%) in the figures and results sections. The significance of differences was accepted at a *p* < 0.05.

## 3 Results

### 3.1 Acidic saline injections into the jaw muscles induce cFos activity in Vc in rats and mice

**Figure 2:**
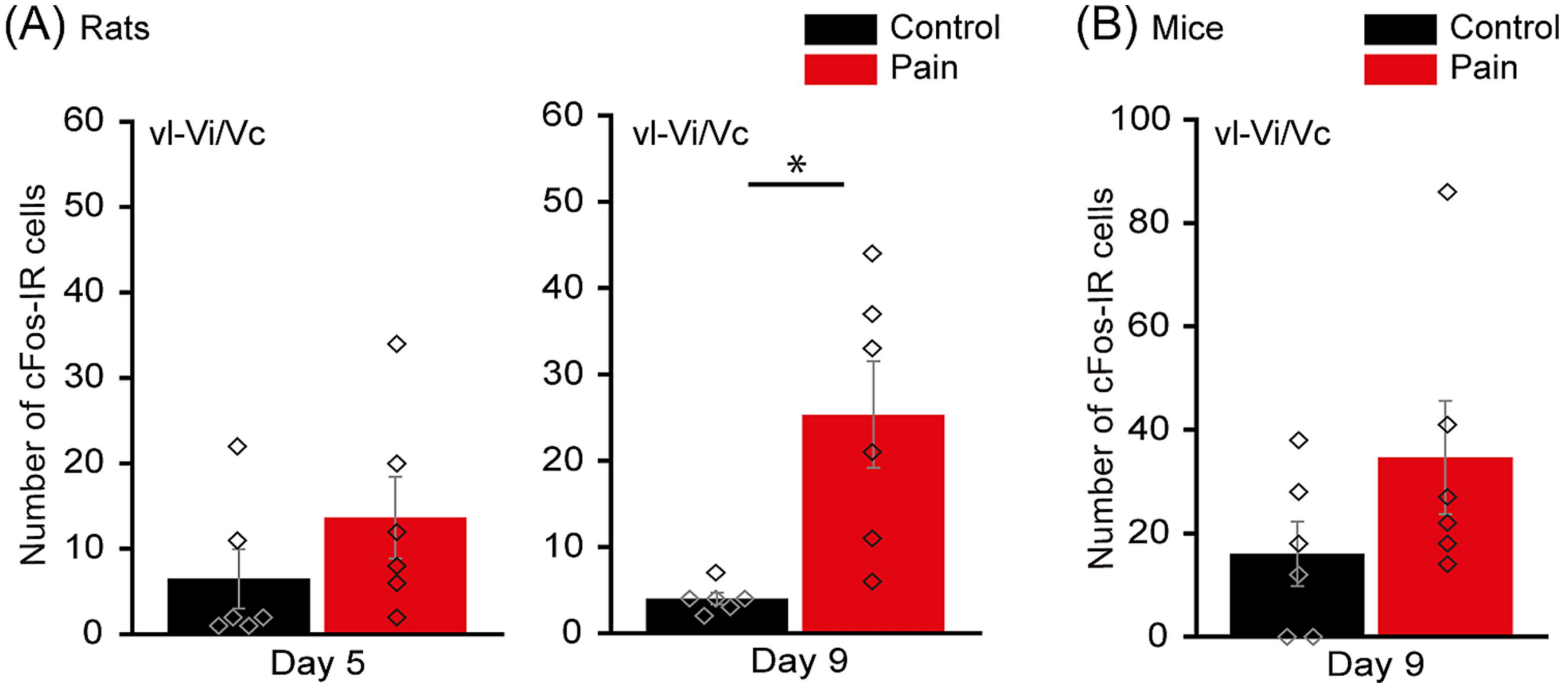
Acidic saline injections increase the number of c-Fos-IR cells in the vl-Vi/Vc. **(A)** Bar chart comparing the number of c-Fos-IR cells in the vl-Vi/Vc of rats, 5 (left panel) and 9 (right panel) days after the 2^nd^ injection in their masseter muscles, in the CTL (black) and PAIN (red) groups. The PAIN group showed more cFos -IR cells than the CTL group at 9 days after the 2^nd^ injection. **(B)** Bar chart comparing the number of c-Fos-IR cells in the vl-Vi/Vc of wild-type mice, 9 days after the 2^nd^ injection in their masseter muscles, in the CTL (black) and PAIN (red) groups. Data are represented as mean ± SEM. Diamonds indicate individual data. ^*^p < 0.05 for comparisons between CTL and PAIN groups, Welch’s *t*-test. cFos-IR, cellular oncogene fos-immunoreactive; vl-Vi/Vc, ventrolateral pole of the subnucleus interpolaris/caudalis transition.

Our previous work suggested that the long-lasting hyperexcitability of NVmes neurons that paralleled the acid-induced mechanical hypersensitivity could lead to activation of nociceptors free endings within the capsule in the muscle (Lund et al., 2010). Here, to further validate the model, we first tested, using immunohistochemistry against the activity marker cFos, whether acidic-saline injections led to an increase of activity of neurons in the area of the trigeminal spinal nucleus known to be associated to orofacial muscle pain (vl-Vi/Vc) (Wang et al., 2006; Nakatani et al., 2018). This area, located ventrolaterally at the junction of the subnucleus interpolaris and the subnucleus caudalis, is known to receive inputs from nociceptors but not from jaw closing MSA. We assessed cFos expression in rats on days 5 and 9 after the 2nd injection. At 5 days post-injection, the number of cFos-IR cells in the PAIN group is slightly higher than that of the CTL group, although there is no statistical difference (CTL group: 6.5 ± 7.8, PAIN group: 13.7 ± 10.7; Mann–Whitney U test, *p* = 0.18; Figure 2A left). The trend is maintained, and the difference becomes significant at 9 days post-injection (CTL group: 4.0 ± 1.5, PAIN group: 25.3 ± 13.8; Welch’s *t*-test, *p* = 0.02; Figure 2A right). Similar findings were obtained in mice 9 days post-injection, although the difference did not reach significance (CTL group: 16.0 ± 13.9, PAIN group: 34.7 ± 24.5; Mann–Whitney U test, *p* = 0.18; Figure 2B).

### 3.2 Acidic saline injections within the jaw muscles cause astrocytes to become reactive in NVmes but not in Vc in rats and mice

**Figure 3:**
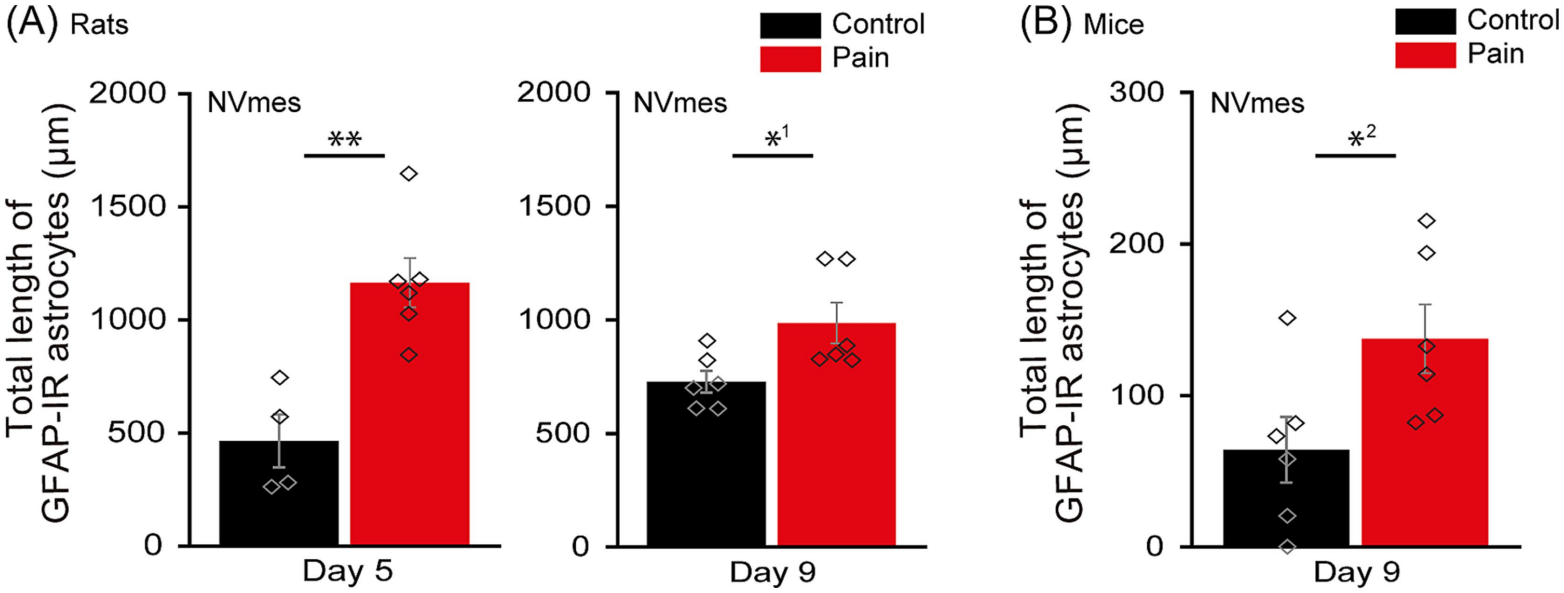
NVmes astrocytes become reactive following injections of acidic saline in the masseter of rats and mice. **(A)** Bar chart comparing the total length of GFAP-IR astrocytes within the ROI in the NVmes of rats, 5 (left panel) and 9 (right panel) days after the 2^nd^ injection in their masseter muscles, in the CTL (black) and PAIN (red) groups. ROI was set as a 150 μm x 400 μm rectangle (see Figure 1). **(B)** Bar chart comparing the total length of GFAP-IR astrocytes within the ROI in the NVmes of Wild-type mice, 9 days after the 2^nd^ injection in their masseter muscles, in the CTL (black) and PAIN (red) groups. ROI was set as a 60 μm x 250 μm rectangle. Data are represented as mean ± SEM. Diamonds indicate individual data. ^*1^*P* < 0.05 for comparisons between CTL and PAIN groups by Student’s *t*-test. ^**^p < 0.01 for comparisons between CTL and PAIN groups by Student’s *t*-test. ^*2^*P* < 0.05 for comparisons between CTL and PAIN groups by Mann–Whitney U test. GFAP-IR, glial fibrillary acidic protein-immunoreactive; NVmes, trigeminal mesencephalic nucleus; ROI, region of interest.

**Figure 4:**
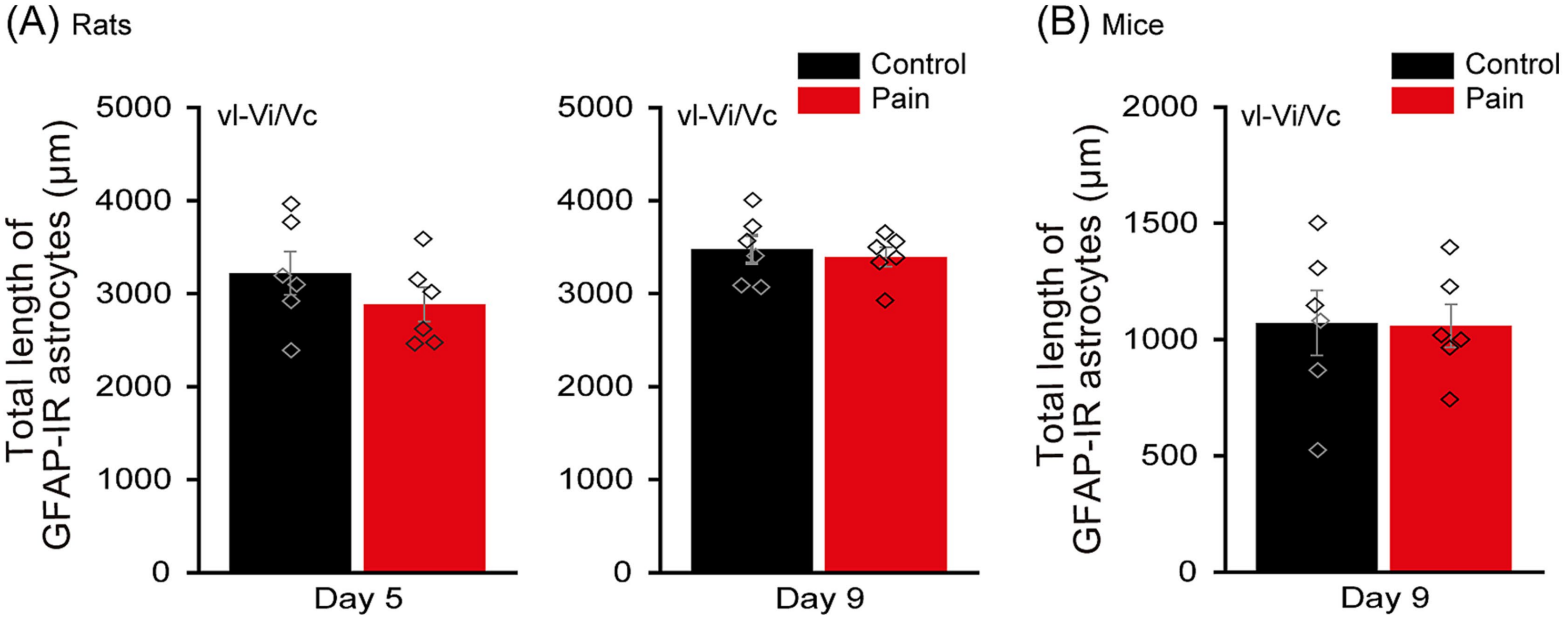
vl-ViVc astrocytes do not become reactive following injections of acidic saline in the masseters of rats and mice. **(A)** Bar chart comparing the total length of GFAP-IR astrocytes within the ROI in the vl-ViVc of rats, 5 (left panel) and 9 (right panel) days after the 2^nd^ injection in their masseter muscles, in the CTL (black) and PAIN (red) groups. ROI was set as a 312 μm × 312 μm square (see Figure 1). **(B)** Bar chart comparing the total length of GFAP-IR astrocytes within the ROI in the vl-ViVc of wild-type mice, 9 days after the 2^nd^ injection in their masseter muscles, in the CTL (black) and PAIN (red) groups. ROI was set as a 198 μm × 198 μm square. Data are represented as mean ± SEM. Diamonds indicate individual data. There were no significant differences between the control and pain groups across all conditions. GFAP-IR, glial fibrillary acidic protein-immunoreactive; ROI, region of interest; vl-Vi/Vc, ventrolateral pole of the subnucleus interpolaris/caudalis transition.

The origin and mechanisms underlying the increased excitability and ectopic firing observed in large diameter primary afferent neurons in association to pathological pain are still unidentified (Sas et al., 2023). Since in many pain models, astrocytes have been shown to become reactive (Okada-Ogawa et al., 2009; Borghi et al., 2023; Ahmed et al., 2025) and having demonstrated that optogenetic stimulation of neighboring astrocytes causes firing in NVmes neurons in GFAP-ChR2-EYFP mice (Gaudel et al., 2025), we then sought to determine if astrocytes also become reactive in our acidic saline injection-induced chronic jaw muscle pain model. Total length of GFAP-IR astrocytes in NVmes and vl-Vi/Vc was evaluated at post-injection 5 and 9 days in rats, and post-injection day 9 in mice. The total length of GFAP-IR astrocytes of the PAIN group is significantly larger than that of the CTL group at 5 (CTL group: 464.9 ± 203.1 μm, PAIN group: 1164.8 ± 243.5 μm; Student’s *t*-test, *p* = 0.003; Figure 3A left) and 9 days (CTL group: 727.8 ± 108.0 μm, PAIN group: 986.5 ± 200.1 μm; Mann–Whitney U test, *p* = 0.04; Figure 3A right) in rats and at 9 days post-injection in mice (CTL group: 64.1 ± 48.3 μm, PAIN group: 137.4 ± 50.8 μm; Student’s *t*-test, *p* = 0.04; Figure 3B). In contrast to NVmes, total length of GFAP-IR astrocytes in vl-Vi/Vc did not differ between CTL and PAIN groups in both rats (Day5: CTL group: 3220.9 ± 524.1 μm, PAIN group: 2885.1 ± 408.3 μm; Student’s *t*-test, *p* = 0.29; Day9: CTL group: 3477.2 ± 333.9 μm, PAIN group: 3394.9 ± 234.2 μm; Student’s *t*-test, *p* = 0.66; Figure 4A left and right, respectively) and mice (CTL group: 1071.6 ± 312.7 μm, PAIN group: 1058.8 ± 207.0 μm; Student’s *t*-test, *p* = 0.94; Figure 4B).

### 3.3 Acidic saline injections within the jaw muscles increase the excitability of NVmes neurons in mice

**Figure 5:**
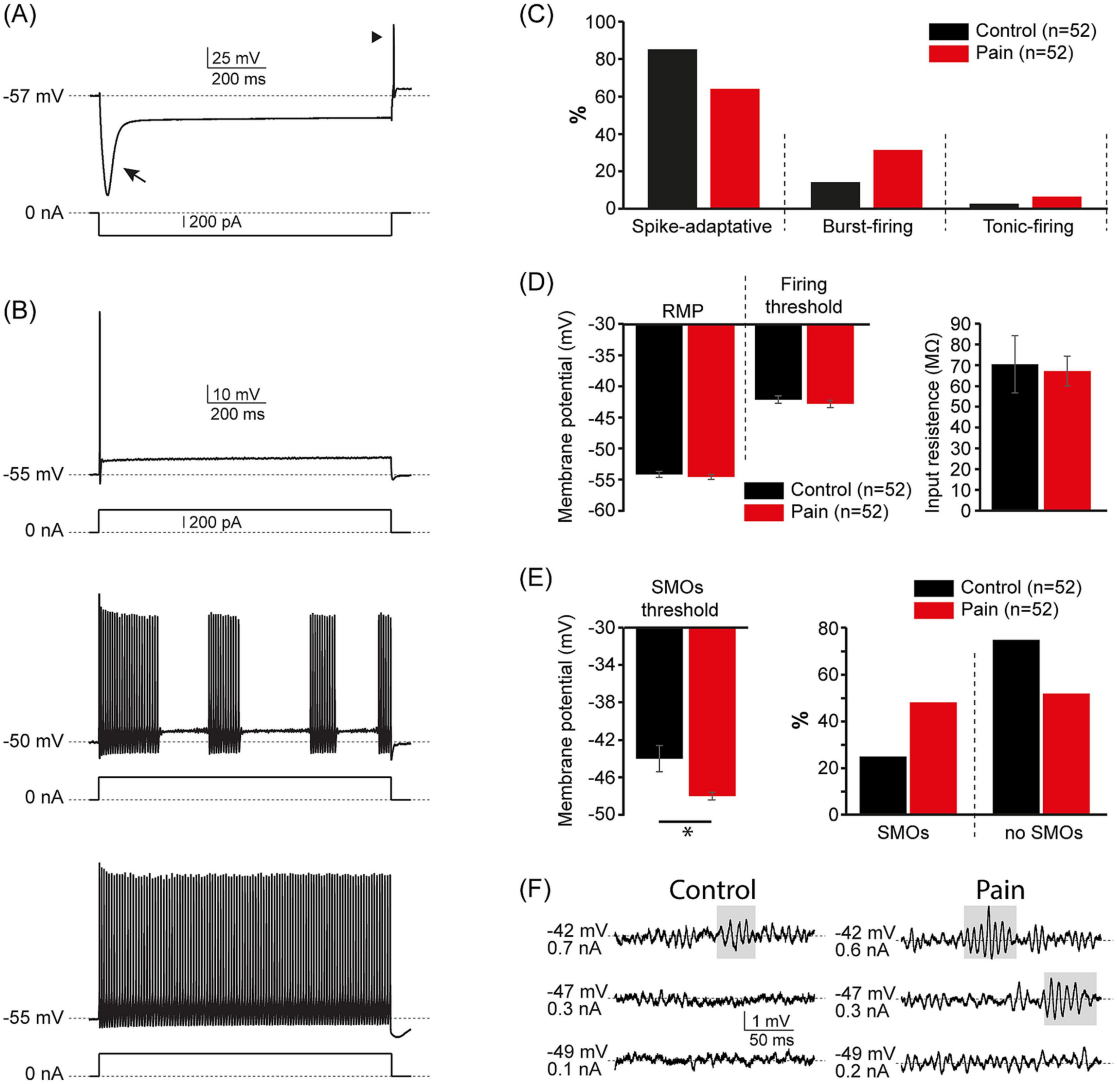
Acidic saline injections augment NVmes neurons excitability by decreasing the SMOs threshold and by increasing their incidence. **(A)** Membrane response (top trace) of an NVmes neuron to injection of a hyperpolarizing current pulse (bottom trace). The arrow and arrowhead point the sag and the rebound action potential, respectively. **(B)** Membrane responses of a spike-adaptative (top), a burst-firing (middle), and a tonic-firing (bottom) NVmes neuron. **(C)** Bar chart of the relative distribution of the firing profiles of the NVmes neurons in the control (black) and pain (red) groups. **(D)** Bar chart comparing the RMP, the firing voltage threshold (left) and the input resistance (right) of the NVmes neurons from the control (black) and pain (red) groups. **(E)** Left: bar chart comparing the SMOs threshold of the NVmes neurons from the control (black) and pain (red) groups. Right: bar chart of the relative distribution of the oscillating and non-oscillating neurons in the control (black) and pain (red) groups. **(F)** Membrane responses of an NVmes neuron from the control group (left) and an NVmes neuron from the pain group (right) showing the emergence of SMOs at a more hyperpolarized potential in the neuron from the pain group. The membrane potential and the amount of injected current are given on the left of each trace. Data in **(D,E)** are represented as mean ± SEM. *P < 0.05, Student *t*-test. NVmes, trigeminal mesencephalic nucleus; RMP, resting membrane potential; SMOs, subthreshold membrane oscillations.

Using whole cell patch-clamp recordings, we then test whether NVmes neurons in mice display increased excitability, as in rats, following the same bilateral injection protocol of either normal (CTL group) or acidic (PAIN group) saline within their masseters (Table 1). As in Lund et al. (2010), the animals were sacrificed at various times (1–26 days) following the second injection. One hundred and four neurons recorded in the NVmes nucleus of 23 WT mice and 21 GFAP-ChR2-EYFP mice fulfilled our inclusion criteria. Those neurons were equally distributed between the control and the experimental groups (*n =* 52 each). They were filled with Alexa Fluor (488 or 594), and all showed the typical pseudo-unipolar morphology of primary sensory afferents. The recorded neurons also showed the typical electrophysiological signature of NVmes neurons consisting of a prominent sag caused by a strong inward rectification upon membrane hyperpolarization (Figure 5A), and their responses to membrane depolarization could still be classified in three distinct firing patterns: spike-adaptative, burst and tonic-firing (Figure 5B, top, middle and bottom traces, respectively). However, there was a significant between-group difference in the distribution of firing patterns (Exact Fisher test, *p* < 0.001) where a significantly greater percentage of neurons from the PAIN group were of the bursting type as shown in Figure 5C. Additionally, all but one neuron from the PAIN group (98%), fired a rebound action potential at the offset of the hyperpolarizing pulses (Figure 5A, arrowhead) while only 43 (83%) of the control neurons displayed this behavior. The basic electrophysiological characteristics of the recorded neurons are summarized in Table 3. There were no statistical differences in the resting membrane potential (RMP), the firing threshold, and the input resistance between neurons from the CTL and PAIN groups (Figure 5D and Table 3). Only the threshold of the voltage-dependent SMOs was found to be significantly more hyperpolarized in the PAIN relative to the CTL group (CTL group: −44 ± 1.4 mV, PAIN group: −48 ± 0.7 mV; Student *t*-test, *p* = 0.01; Figure 5E left and Figure 5F). There were also significantly more neurons in the PAIN group that exhibited SMOs (CTL group: 25%, PAIN group: 48%; *X^2^* = 5.971, df = 1, *p* = 0.01; Figure 5E right).

**Table 3:** Electrophysiological characteristics of NVmes neurons.

Electrophysiological characteristics	WT + GFAP-ChR2-EYFP mice *n =* 104		S100β KO mice *n =* 12	
	CTL (*n =* 52)	PAIN (*n =* 52)	CTL (*n =* 6)	PAIN (*n =* 6)
RMP (mV)	−54 ± 0.4^a^	−54 ± 0.4^a^	−54 ± 1.1^b^	−53 ± 1.3^b^
Firing threshold (mV)	−42 ± 0.6^a^	−43 ± 0.5^a^	−45 ± 1.3^b^	−44 ± 0.9^b^
Input resistance (MΩ)	70 ± 14^a^	67 ± 7^a^	61 ± 9^b^	79 ± 23^b^
SMOs threshold (mV)	−44 ± 1.5*	−48 ± 0.7*	−49 ± 1.5^b^	−50 ± 1.1^b^

Values are mean ± SEM. ^a^*P* >0.05 for comparisons CTL and PAIN groups for the WT/GFAP-ChR2-EYFP mice, Student *t*-test. ^b^*P* > 0.05 for comparisons CTL and PAIN groups for the S100β KO mice, Student *t*-test. *p = 0.01 for comparisons CTL and PAIN groups for the WT/GFAP-ChR2-EYFP mice, Student *t*-test. ChR2, channelrhodopsin 2; EYFP, enhanced yellow fluorescent protein; GFAP, glial fibrillary acidic protein; KO, knock-out; NVmes, trigeminal mesencephalic nucleus; n, number of neurons; RMP, resting membrane potential; SMOs, subthreshold membrane oscillations; WT, wild type.

**Figure 6:**
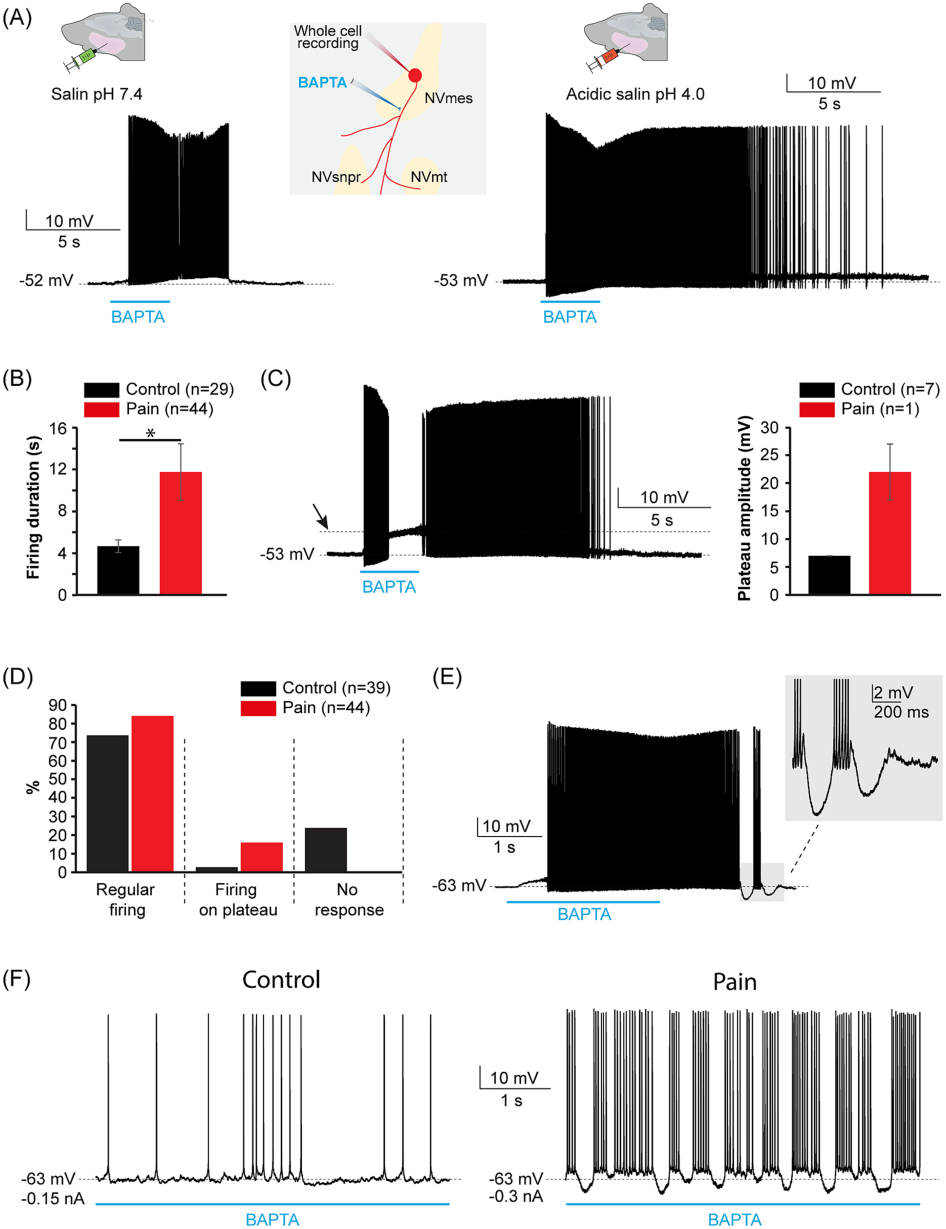
Chelation of extracellular calcium along the axons of NVmes neurons produces more robust firings in the acidic saline injected mice. **(A)** Applications of the Ca^2+^ chelator BAPTA along the axonal process of NVmes neurons produce a more robust firing in neurons from the pain group (right) than in those from the control group (left). Middle inset: Cartoon illustrating the experimental set-up. **(B)** Bar chart comparing the duration of the BAPTA-induced firing in NVmes neurons from the control (black) and pain (red) groups. **(C)** Left: Example of a BAPTA-induced firing response superimposed on a plateau potential in an NVmes neuron from the pain group. The arrow shows the amplitude of the underlying plateau. Right: Bar chart comparing the amplitude of the plateau potential in the BAPTA-induced firing in the NVmes neurons from the control (black) and pain (red) groups. **(D)** Bar chart of the relative distribution of the neuronal responses to the axonal applications of BAPTA in the control (black) and pain (red) groups. **(E)** Example of a BAPTA-induced firing showing bursts on plateau in a NVmes neuron from the pain group. **(F)** Right: Example of a BAPTA-induced firing showing bursts on plateau in a NVmes neuron from the pain group upon membrane hyperpolarization. Left: No burst was elicited by membrane hyperpolarization in the neuron from the control group. Data in **(B,C)** are represented as mean ± SEM. *P < 0.05, Student *t*-test. NVmes, trigeminal mesencephalic nucleus; NVmt, trigeminal motor nucleus; NVsnpr, trigeminal main sensory nucleus.

BAPTA was locally applied along the axons of the recorded neurons, as was done in Gaudel et al. (2025) to test whether the group of treatment influences the firing typically induced by BAPTA in those neurons (Gaudel et al., 2025). Forty-four applications were made at 31 positions along the axon of 19 neurons (*n =* 19) from the PAIN group, and 38 applications were made at 28 positions along the axon of 18 neurons (*n =* 18) from the CTL group (Table 4). The duration (CTL group: 5 ± 0.4 s, PAIN group: 6 ± 1.1 s; Mann–Whitney U test, *p* = 0.4) and position (CTL group: 83 ± 9 μm, PAIN group: 75 ± 7 μm; Student *t*-test, *p* = 0.5) of the application along the axon of the recorded neurons were equivalent between both groups. Firing was induced by all the applications in the PAIN group at a latency of 1.4 ± 0.2 s and by 29 applications of the 38 applications in the CTL group at a latency of 1.5 ± 0.2 s (Student *t*-test, *p* = 0.7; Table 4). Figure 6A illustrates an example of firing from a neuron of each group in response to BAPTA application. The duration of response to BAPTA application was significantly higher in the PAIN group than in the CTL group (CTL group: 5 ± 1 s, PAIN group: 12 ± 3 s; Mann–Whitney U test, *p* = 0.03; Figure 6B). In 7 cases of the application from the PAIN group, the firing was superimposed on a large amplitude depolarizing block (22 ± 5 mV; Figures 6C,D) while only one firing response to BAPTA application was superimposed on such a plateau of 7 mV in the CTL group. Another peculiarity of the firing in response to axonal BAPTA application was the presence of bursts on plateau as shown in Figure 6E, at RMP or upon membrane hyperpolarization (Figure 6F right) in 6 neurons (*n =* 6) of the PAIN group which was never observed (*n =* 0) in the firing responses of the control group (Figure 6F left).

**Table 4:** Effects of axonal BAPTA applications on NVmes neurons.

Mice	Group	Position along the axon (μm)	Application duration (s)	Firing		No firing
				Latency (s)	Duration (s)	
	(*n =* number of neurons)	(*n =* number of positions)	(*n =* number of applications)	(*n =* number of responses)		(*n =* number of responses)
WT mice and GFAP-ChR2-EYFP mice	CTL	83 ± 9^b^	6 ± 1.1^a^	1.5 ± 0.2^b^	5 ± 1*	
	(18)	(28)	(38)	(29)		(9)
	PAIN	75 ± 7^b^	5 ± 0.4^a^	1.4 ± 0.2^b^	12 ± 3*	
	(19)	(31)	(44)	(44)		
S100β KO mice	CTL	66 ± 17^c^	6 ± 1^c^	2.1 ± 0,4^c^	11 ± 5^c^	
	(3)	(3)	(9)	(9)		
	PAIN	73 ± 21^c^	6 ± 1^c^	1.4 ± 0.3^c^	6 ± 1^c^	
	(3)	(3)	(9)	(9)		

Values are mean ± SEM. Some neurons were tested at multiples sites along the axons and for different durations. ^a^*P* > 0.05 for comparisons neutral and acidic saline for the WT/GFAP-ChR2-EYFP mice; Mann–Whitney U test. ^b^*P* > 0.05 for comparisons neutral and acidic saline for the S100β KO mice, Student *t*-test. *P = 0.03 for comparisons neutral and acidic saline for the WT/GFAP-ChR2-EYFP mice, Mann–Whitney U test. ChR2, channelrhodopsin 2; EYFP, enhanced yellow fluorescent protein; GFAP, glial fibrillary acidic protein; KO, knock-out; NVmes, trigeminal mesencephalic nucleus; WT, wild type.

### 3.4 Astrocytes play a role in chronic pain

To determine whether the acid-induced astrocytic reactivity observed above translates into a difference in the ability of astrocytes to influence the excitability of NVmes neurons, we repeated the neutral and acidic saline injections in the masseters of GFAP-ChR2-EYFP mice (Table 1). For this experiment, successful whole-cell recordings were obtained from 9 animals (*N =* 9) in the PAIN group and from 7 animals (*N =* 7) in the CTL group. The recorded neurons were filled with Alexa Fluor 594 through the patch pipette to visualize their soma and axon and to allow manual delimitation of defined zones for optogenetic stimulation of neighboring astrocytes. The parameters of the photostimulated areas were measured offline and were equivalent between both groups (Table 5).

**Table 5:** Stimulated areas.

Group	Surface	Distance from the soma	Length along the axon
	(μm^2^)	(μm)	(μm)
CTL	12,150 ± 4,060^a^ *n =* 7	39 ± 15^a^	50 ± 9^b^
PAIN	6,939 ± 1,566^a^ *n =* 10	78 ± 37^a^	48 ± 9^b^

Values are mean ± SEM. *n =* number of images analyzed. ^a^*P* > 0.05 for comparisons CTL and PAIN groups, Mann–Whitney U test. ^b^*P* > 0.05 for comparisons CTL and PAIN groups, Student t test.

**Figure 7:**
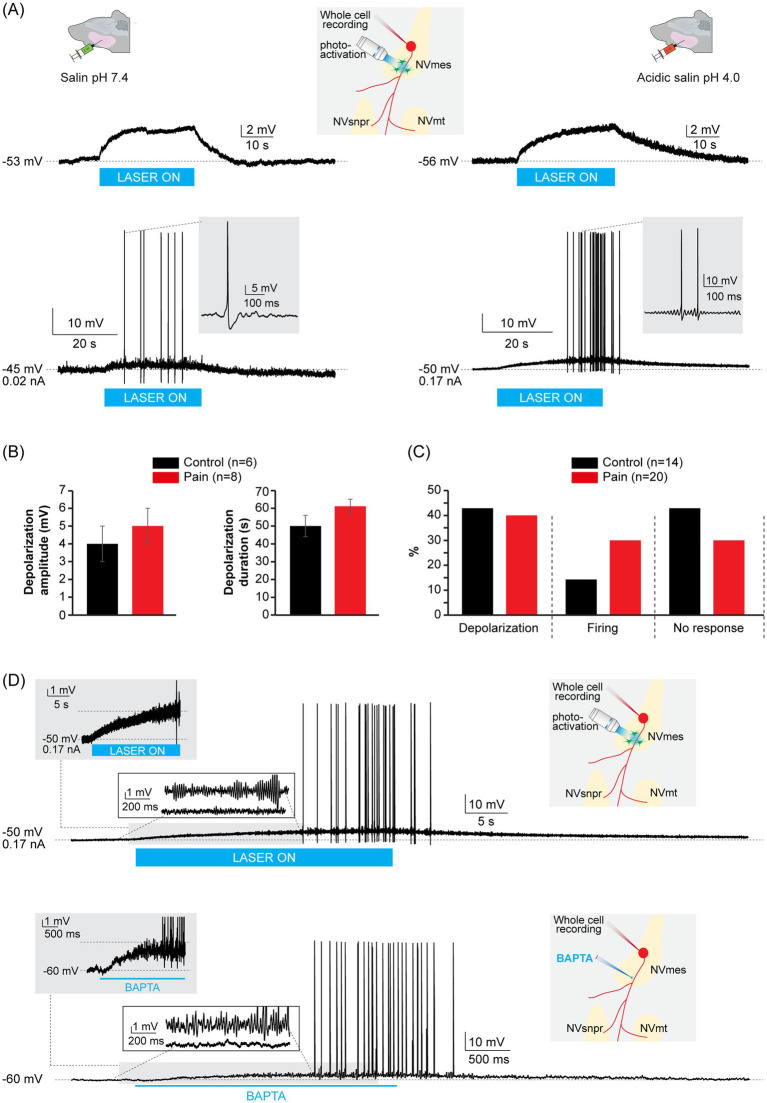
Responses to optogenetic stimulation of peri-axonal astrocytes in GFAP-ChR2-EYFP mice do not differ between the neutral and the acidic saline injected groups. **(A)** Example of long-lasting depolarization responses (top traces) elicited in NVmes neurons by optogenetic stimulation of peri-axonal astrocytes from the control (left) and pain (right) groups. **(A)** Example of firing responses (bottom traces) elicited in NVmes neurons by optogenetic stimulation of peri-axonal astrocytes from the control (left) and pain (right) groups. Insets: Cartoons illustrating the experimental set-up. **(B)** Bar chart comparing the amplitude (left) and duration (right) of the long-lasting depolarization elicited by optogenetic stimulation of peri-axonal astrocytes in NVmes neurons from the control (black) and pain (red) groups. **(C)** Bar chart of the relative distribution of the neuronal responses to the optogenetic stimulation of peri-axonal astrocytes in NVmes neurons from the control (black) and pain (red) groups. **(D)** Examples of firing induced by optogenetic stimulation of peri-axonal astrocytes (top) and axonal application of BAPTA (bottom) in a NVmes neuron showing the similarities between both responses: the underlying depolarization (left shaded insets), the emergence of SMOs prior to the firing (left non-shaded insets). Right insets: Cartoons illustrating the experimental set-ups. Data in **(B)** are represented as mean ± SEM. ChR2, channelrhodopsin 2; EYFP, enhanced yellow fluorescent protein; GFAP, glial fibrillary acidic protein; NVmes, trigeminal mesencephalic nucleus; NVmt, trigeminal motor nucleus; NVsnpr, trigeminal main sensory nucleus.

Optogenetic activation of the astrocytes surrounding the axonal process of 7 neurons (*n =* 7) from the CTL group at their RMP produced no response in 3 cells (*n =* 3), and a long-lasting depolarization at a latency of 0.3 ± 0.1 s in the 4 remaining cells (*n =* 4) (Figure 7A left, top trace; Table 6). Upon membrane depolarization, optogenetic stimulation produced no response in 3 cells (*n =* 3), a long-lasting depolarization at a latency of 0.1 ± 0.07 s in 2 cells (*n =* 2) and firing emerging from the long-lasting depolarization evoked at RMP in 2 cells (*n =* 2) at latencies of 4 s and 19 s (Figure 7A left, bottom trace; Table 6).

**Table 6:** Effects of optogenetic stimulation of astrocytes on NVmes neurons.

Group	Condition	Depolarization			Firing		No effect
		Latency	Amplitude	Duration	Latency	Duration	
		(s)	(mV)	(s)	(s)	(s)	
CTL	At RMP	0.3 ± 0.1 (*n =* 4) *n =* 4	4 ± 0.8	52 ± 7			(*n =* 3) *n =* 3
	With depolarization	0.1 ± 0.07 (*n =* 2) *n =* 2	5 ± 1.5	53 ± 10	11 ± 7 (*n =* 2) *n =* 2	23 ± 3	(*n =* 3) *n =* 3
PAIN	At RMP	0.2 ± 0.1 (*n =* 5) *n =* 5	6 ± 1.5	57 ± 4	10 (*n =* 1) *n =* 1	6	(*n =* 4) *n =* 4
	With depolarization	0.7 ± 0.4 (*n =* 3) *n =* 3	4 ± 0.3	69 ± 7	12 ± 3.5 (*n =* 5) *n =* 5	26 ± 7	(*n =* 2) *n =* 2
CTL	RMP + depolarization	0.2 ± 0.1^a^ (*n =* 6)	4 ±1^a^	50 ± 6^a^	11 ± 7 (*n =* 2)	23 ± 3	(*n =* 6)
PAIN	RMP + depolarization	0.2 ± 01^a^ (*n =* 8)	5 ± 1^a^	61 ± 4^a^	13 ± 4 (*n =* 6)	21 ± 7	(*n =* 6)

Values are mean ± SEM for data that can be averaged; exact values for *n* < 3. ^a^*P* > 0.05 for comparisons CTL and PAIN groups, Student *t*-test. (n), number of stimulation sites; n, number of neurons; NVmes, trigeminal mesencephalic nucleus; RMP, resting membrane potential.

Optogenetic activation of the astrocytes surrounding the axonal process of 10 neurons (*n =* 10) from the pain group at their RMP produced no response in 4 cells (*n =* 4), a long-lasting depolarization at a latency of 0.2 ± 0.1 s in 5 other cells (*n =* 5) (Figure 7A right, top trace; Table 6) and firing in the remaining cell at a latency of 9.7 s. Upon membrane depolarization, optogenetic stimulation produced no response in 2 cells (*n =* 2), a long-lasting depolarization at a latency of 0.2 ± 0.1 s in 3 other cells (*n =* 3), firing in 3 neurons (*n =* 3) (Figure 7A right, bottom trace) and increased the frequency of the depolarization-induced firing in 2 other neurons (*n =* 2) (0.4 ± 0.3 Hz to 0.8 ± 0.1 Hz). The increased frequency responses were pooled with the firing responses. The firings occurred at a latency of 12 ± 3.5 s and lasted 26 ± 7 s (Table 6).

For comparison between both groups, the responses at RMP and with membrane depolarization are pooled (Table 6). The latency (CTL group: 0.2 ± 1 s, PAIN group: 0.2 ± 1 s; Student *t*-test, *p* = 0.5), amplitude (CTL group: 4 ± 1 mV, PAIN group: 5 ± 1 mV; Student *t*-test, *p* = 0.2) and duration (CTL group: 50 ± 6 s, PAIN group: 61 ± 4 s; Student *t*-test, *p* = 0.06) of the long-lasting depolarizations were equivalent between both groups (Figure 7B; Table 6). Figure 7C shows the distribution of the effects of the optogenetic stimulations of peri-axonal astrocytes on the NVmes neurons. There was no significant between-group difference in the distribution of the responses of the NVmes neurons to the optogenetic stimulation of the surrounding astrocytes (Exact Fisher test, *p* = 0.607).

In 7 cells (CTL group: *n =* 3, PAIN group: *n =* 4) where both BAPTA axonal applications and optogenetic stimulations of the peri-axonal astrocytes were performed, the responses to the optogenetic stimulation clearly mimic some aspects of the responses to axonal BAPTA application. The example shown in Figure 7D shows that both stimuli elicited membrane depolarization (top and bottom gray insets) and the occurrence of high-amplitude SMOs (top and bottom non-shaded insets) that trigger the firing episodes (top and bottom traces). The similarities encountered in the responses to both stimuli support our hypothesis that the effects of the astrocytic optogenetic stimulation involve calcium chelation in the extracellular environment surrounding the axonal processes of these neurons.

**Figure 8:**
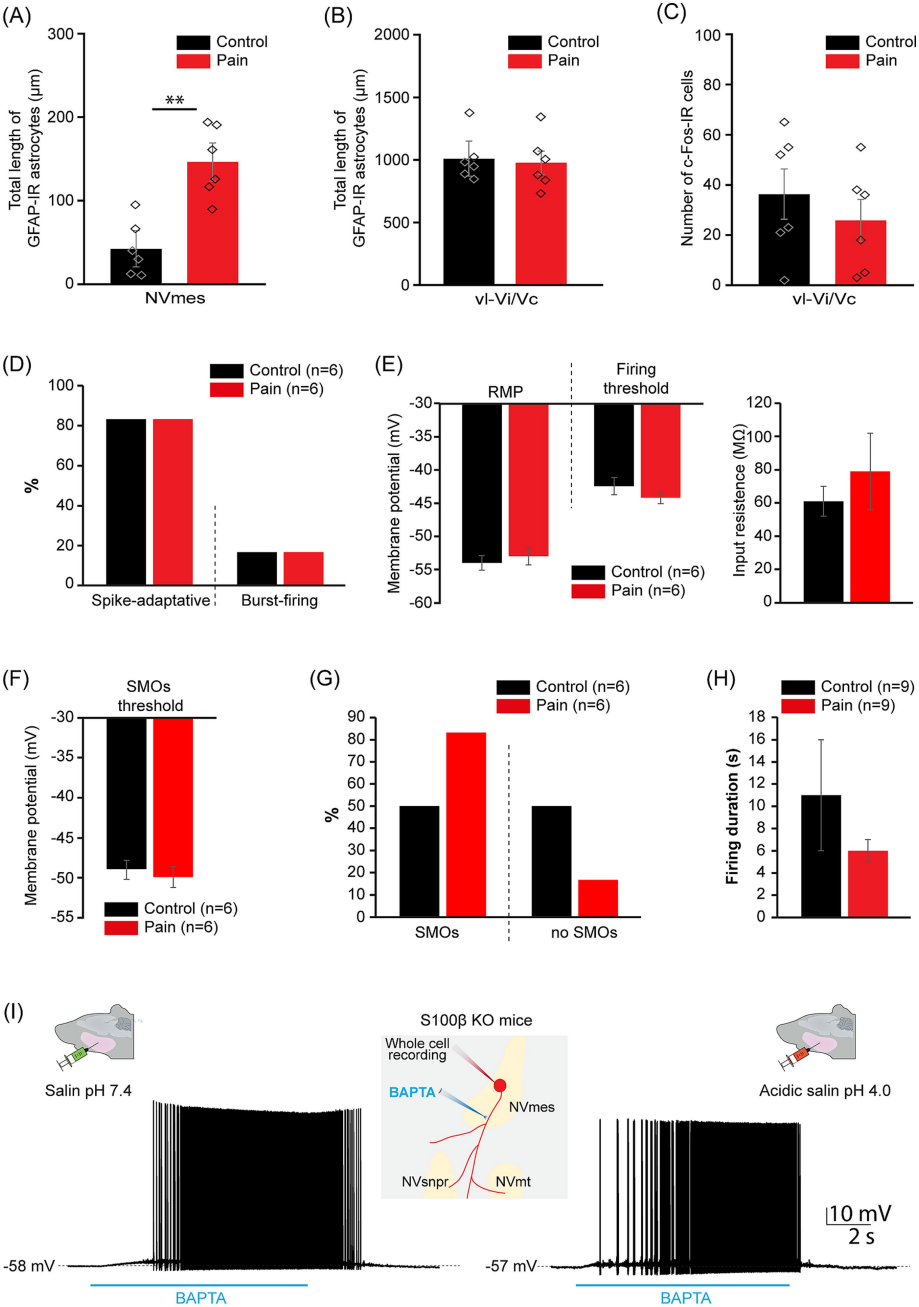
Acidic saline injections cause NVmes astrocytes to become reactive but do not alter the excitability of NVmes neurons in S100β KO mice. **(A)** Bar chart comparing the total length of GFAP-IR astrocytes in the NVmes of S100β KO mice, 9 days after 2^nd^ injection into their masseter muscles, in the CTL (black) and PAIN (red) groups. **(B)** Bar chart comparing the total length of GFAP-IR astrocytes in the vl-Vi/Vc of S100β KO mice, 9 days after 2^nd^ injection into their masseter muscles, in the CTL (black) and PAIN (red) groups. **(C)** Bar chart comparing the number of c-Fos-IR cells in the vl-Vi/Vc of S100β KO mice, 9 days after 2^nd^ injection into their masseter muscles, in the CTL (black) and PAIN (red) groups. **(D)** Bar chart of the relative distribution of the neuronal firing responses to depolarizing pulses in the control (black) and pain (red) groups of the S100β KO mice. **(E)** Bar chart comparing the RMP, the firing voltage threshold (left) and the input resistance (right) of the NVmes neurons from the control (black) and pain (red) groups of the S100β KO mice. **(F)** Bar chart comparing the SMOs threshold of the NVmes neurons from the control (black) and pain (red) groups of the S100β KO mice. **(G)** Bar chart of the relative distribution of the oscillating and non-oscillating neurons in the control (black) and pain (red) groups of the S100B KO mice. **(H)** Bar chart comparing the duration of the BAPTA-induced firing in NVmes neurons from the control (black) and pain (red) groups of the S100β KO mice. **(I)** Applications of the Ca^2+^ chelator BAPTA along the axonal process of NVmes neurons produce similar firing in the pain (right) and control groups (left). Middle inset: Cartoon illustrating the experimental set-up. Data in **(A–C,E,F,H)** are represented as mean ± SEM. Diamonds in **(A–C)** indicate individual data. ^**^p < 0.01 for comparison between CTL and PAIN groups, Student’s *t*-test. c-Fos-IR, cellular oncogene fos-immunoreactive; GFAP-IR, glial fibrillary acidic protein-immunoreactive; KO, knock-out; NVmes, trigeminal mesencephalic nucleus; NVmt, trigeminal motor nucleus; NVsnpr, trigeminal main sensory nucleus; RMP, resting membrane potential; ROI, region of interest; SMOs, subthreshold membrane oscillations; vl-Vi/Vc, ventrolateral pole of the subnucleus interpolaris/caudalis transition.

Our previous work suggested that S100β is the likely candidate to mediate this modulatory effect on the NVmes neurons’ excitability. We then sought to examine how the genetic deletion of S100β interferes with the acidic saline-induced jaw muscle chronic pain. At first, we compared the expression of GFAP and cFos in NVmes and vl-Vi/Vc of S100β KO mice that received injections of either neutral or acidic saline. As was the case for WT mice and rats, the total length of GFAP-IR astrocytes was greater in the NVmes of the PAIN group (CTL group: 42.3 ± 30.0 μm, PAIN group: 146.3 ± 38.6 μm; Student *t*-test, *p* = 0.001; Figure 8A) while no differences was found between groups for GFAP-IR astrocytes in vl-Vi/Vc (CTL group: 1010.0 ± 173.2 μm, PAIN group: 977.4 ± 196.7 μm; Student *t*-test, *p* = 0.79; Figure 8B). However, in contrast to WT mice and rats, there was no difference in the expression of cFos in vl-Vi/Vc of S100β KO mice receiving different treatments (CTL group: 36.3 ± 22.4, PAIN group: 25.8 ± 18.8; Student *t*-test, *p* = 0.44; Figure 8C).

We then test whether the NVmes neurons in S100β KO mice displayed the same increased excitability with acidic saline injection in the masseter muscles than rats and WT and GFAP-ChR2-EYFP mice. Twelve NVmes neurons (*n =* 12) were recorded in S100β KO mice that were equally distributed between both groups. No significant differences could be found in any of the cell properties (Table 3; Figures 8D–F) between both groups. Even the higher number of cells with SMOs in the pain group relative to control was not significant (Exact Fisher test, *p* = 0.273; Figure 8G). Responses to BAPTA were also tested at 3 positions along the axons of 3 neurons (*n =* 3) in each group. The duration (CTL group: 6 ± 1 s, PAIN group: 6 ± 1 s; Student *t*-test, *p* = 0.5) and position (CTL group: 66 ± 17 μm, PAIN group: 73 ± 21 μm; Student *t*-test, *p* = 0.4) of the application along the axon of the recorded neurons were equivalent between both groups (Table 4). A total of nine applications were made at these 3 positions which all produced firing in the recorded neurons from both groups at similar latencies (CTL group: 2.1 ± 0.4 s, PAIN group: 1.4 ± 0.3 s; Student *t*-test, *p* = 0.07; Table 4) and with similar durations (CTL group: 11 ± 5 s, PAIN group: 6 ± 1 s; Student *t*-test, *p* = 0.2; Figures 8H,I). Figure 8I illustrates an example of firing from a neuron of each group in response to BAPTA application.

## 4 Discussion

Using acidic saline-injections in masseter muscles of rodents as a model for chronic jaw muscle pain, this study shows that findings previously obtained in rats with this model were reproduced in mice and that this procedure increases: (1) The number of cFos-IR cells in vl-Vi/Vc, (2) reactivity of NVmes astrocytes (as measured with GFAP-IR) in rats and mice, (3) Excitability of NVmes neurons in mice, as was the case in rats, and robustness of the responses to BAPTA applications (used to mimic S100β). Except for the increased reactivity of astrocytes in NVmes, none of these effects could be reproduced in S100β KO. Collectively, these findings support, but do not directly demonstrate, that a possible sequence of events leading to pain chronicisation in the acidic-induced pain model as formulated by Sas et al. (2023) could be: (1) acidic saline injection into the jaw muscle leads to increased excitability of NVmes neurons and activation of astrocytes which through release of their S100β maintain hyperexcitability of NVmes neurons. (2) The ectopically initiated firing in these neurons could then propagate to their peripheral endings and lead to glutamate release, which (3) could activate nociceptors free endings found nearby within the spindle capsule. (4) These in turn would activate nociceptive pathways in Vc ultimately leading to pain sensation.

In addition to the evidence reported by Bewick et al. (2005), showing that the peripheral endings of proprioceptors release glutamate through synaptic-like vesicles (SLV) and by Lund et al. (2010) showing the presence of nociceptor endings near these glutamate releasing sites, this hypothesis is supported by the facts that patients with jaw muscle pain have increased interstitial glutamate in their masseters (Castrillon et al., 2010) and that injection of glutamate into the jaw muscle in human induces muscle pain (Cairns et al., 2003; Svensson et al., 2003).

### 4.1 Validation of the pain model in rodents

Many studies investigated superficial tissue pain, such as skin, but there are still few studies on deep tissue pain. This may stem from the belief that both pains share similar mechanisms (Mizumura and Taguchi, 2024). However, some evidence argues against this. For instance, it has been reported that muscle and cutaneous afferents show different electrical activities under neuropathic pain (Michaelis et al., 2000), suggesting that the mechanisms in chronic pain might differ. Furthermore, muscle pain is characterized by pain due to a decrease in tissue pH. This is often referred to as “soreness” or “sngception,” a term coined in a more recent literature (Lin et al., 2018; Hung et al., 2023; Han et al., 2025; Lee et al., 2025) and is considered distinct from nociceptive pain. Lee et al. (2025) showed that ASIC3, expressed on proprioceptors innervating muscle spindles in the hindlimb, is significantly involved in this soreness. However, other ASICs may be involved in the case of jaw muscles since according to some studies, MSAs in jaw muscles do not express ASIC3, but other subtypes of ASICs, such as ASIC1b and ASIC2a (Molliver et al., 2005; Nakamura and Jang, 2014). According to Nakamura and Jang (2014), application of acidic extracellular solution (pH 6.5–pH 5) on acutely isolated rat NVmes neurons induces an inward current in a pH-dependent manner in voltage-clamp and depolarization and bursts of action potentials in current-clamp experiments. It is likely that the acidic saline injections in the masseter muscles in our experiment induced massive membrane depolarization in the affected neurons which could then be propagated to the neighboring NVmes neurons through gap junctions. This sudden and intense depolarization and firing can perhaps be the trigger for the astrocytic reactivity observed in the NVmes, which, once established, may contribute to maintaining the hyperexcitability of these sensory neurons. The differential expression of ASICs in the afferences innervating the hindlimb and the jaw muscles point to potential mechanistic variations between hindlimb and orofacial muscle pain.

*In vivo* experiments suggest that ASIC3 play a role in secondary hyperalgesia, which increases response to noxious stimuli outside the site of injury, although other types of ASIC contribute to primary hyperalgesia, which increases response to noxious stimuli at the site of injury (Walder et al., 2010). The fact that NVmes neurons lack ASIC3 (Molliver et al., 2005; Nakamura and Jang, 2014), may complicate accurate pain assessment using von Frey filaments in the orofacial region which could require greater technical proficiency than in the hindlimb region to stimulate a trigger point. This may be further complicated in mice because they have fewer muscle spindles in their masseters, and these seem to be concentrated in a column in the anterior portion of the muscle, perhaps limiting their exposure to the acid-saline injection. Studies using similar DOMS or muscle pain models have utilized both mice (Sluka et al., 2003; Lee et al., 2025) and rats (Sluka et al., 2001; Yokoyama et al., 2007; Lund et al., 2010; Walder et al., 2010; Simonic-Kocijan et al., 2013; Sun et al., 2016; Nguyen et al., 2025), but the orofacial pain field has primarily employed rats. This preference is likely due to the technical difficulty of applying the von Frey test to the orofacial region of mice, as they are less adaptable to the experimental environment for behavioral assessment compared to rats. On the other hand, mice are highly advantageous for exploring neurological mechanisms because of the relative ease of generating knockout and transgenic lines. Therefore, it is essential to establish novel indicators for pain assessment in mice that can serve as alternatives to the von Frey test.

Here, we measured cFos expression in the trigeminal spinal nucleus as a pain indicator. A clinical characteristic of muscle pain is that pain is not felt under normal conditions but is felt during exercise or muscle stimulation (Arendt-Nielsen et al., 2003; Mizumura and Taguchi, 2024). Previous studies have quantified allodynia using von Frey filaments or assessed cFos expression following muscle compression in rats (Sluka et al., 2001; Taguchi et al., 2005; Lund et al., 2010; Lee et al., 2025). Therefore, we applied pressure stimulation to the masseter muscle using von Frey filaments before perfusion fixation. In the study of Lund et al. (2010), allodynia induced by acidic saline could be detected with the 15 g von Frey filament. Thus, we used a 15 g von Frey filament in this study as well to stimulate the muscle mechanically before perfusing the animal for cFos immunohistochemistry. We focused on the vl-Vi/Vc area of the spinal nucleus as it was found to be the most responsive area for jaw muscle inflammatory pain (Wang et al., 2006; Nakatani et al., 2018). Expression of c-Fos was higher in the pain groups, but the difference reached significance for both rats and mice only at 9 days post-injection. This result indicates that the vl-Vi/Vc area is also responsive to the acidic saline injection-induced chronic pain. This area receives nociceptive inputs from small-diameter neurons in the trigeminal ganglion that project to the superficial layers of Vc, followed by ascending axons from Vc to vl-Vi/Vc (Sugiyo et al., 2005; Wang et al., 2006), but no direct projection from the NVmes to the best of our knowledge. It has been reported that some NVmes neurons project to the deep layers of the Vc (Luo et al., 1995), but there is no evidence indicating projections from the deep layers of Vc to the vl-Vi/Vc region. Therefore, the observed increase in cFos-IR cells within the vl-Vi/Vc region is likely mediated by intramuscular nociceptors or by interneurons. Nociceptors in the masseter muscles also have ASIC3 and could be directly activated by the acidic saline injection (Molliver et al., 2005; Gazerani and Cairns, 2018), and some studies suggested that the ASIC3 in nociceptors plays a role in the muscle pain during DOMS in rats (Connor et al., 2005; Matsubara et al., 2019). Although this may contribute in the initial phase of pain, it is unlikely the cause of the later phase since the increase in cFos becomes significant only much later after the injection (after 9 days) well after excitability changes are detected in NVmes, and because genetic deletion of ASIC3 from nociceptors does not prevent the acid-induced hyperalgesia (Lee et al., 2025). Future studies will be required to elucidate the dynamics in the muscle spindle and to further define the role of nociceptors in this chronic jaw muscle pain model, but for the time being our observations lead us to suggest that the hyperexcitability of the NVmes neurons could be an associated change in the earlier stages of pain induction but could become a causal driver for its maintenance by downstream activation of nociceptors.

### 4.2 Potential contribution of NVmes astrocytes to chronic jaw muscle pain

Globally, our electrophysiological findings in mice were consistent with the results reported in rats by Lund et al. (2010), although fewer basic electrophysiological characteristics were significantly affected. This may be due to the smaller sample size in this study or to differences reported in the electrophysiological properties of NVmes neurons in the two species (Dapino et al., 2023). NVmes neurons excitability is mostly regulated by two main currents, the D-type K^+^ current (ID) and I_NaP_, in both rats and mice. However, despite comparable RMP and input resistance, NVmes neurons from mice are less excitable than rat NVmes neurons because of a significantly higher magnitude of ID (Dapino et al., 2023). ID antagonizes I_NaP_ and its higher magnitude (gmax 2.26 ± 0.90 nS/pF vs. 0.12 ± 0.075 nS/pF, ID vs. I_NaP_, respectively) makes it more determinant in setting the NVmes neurons input resistance and RMP (Dapino et al., 2023). The values reported by Dapino et al. (2023) were most likely obtained from spike-adaptative neurons since they are the more abundant type. But ID may be lower in the burst-firing neurons as it has been reported that I_4AP_ is significantly lower in these types of neurons when the membrane was depolarized to −56 mV and above than in the spike-adaptative neurons in rats (Yang et al., 2009). Since ID and I_NaP_ activation and inactivation curves do not completely overlap [V_1/2MAX_ -36.3 ± 4.2 mV vs. − 45.9 ± 4.5 mV, ID vs. I_NAP_, respectively (Dapino et al., 2023)] we suppose that there will be a window of membrane potentials where the antagonizing effect of ID on I_NaP_ would be sufficiently reduced to allow the I_NaP_-mediated effect on the excitability of the NVmes neurons to become apparent which seems to be around the SMOs threshold in our experiments.

The electrophysiological properties of NVmes neurons in mice were consistent with those previously reported by Gaudel et al. (2025). The recorded neurons showed a strong inward rectification producing a prominent sag upon membrane hyperpolarization and SMOs followed by action potential initiation. Current-clamp recordings revealed that in WT mice the percentage of neurons exhibiting burst-firing and tonic-firing patterns significantly increased compared to the CTL group, but notably, this increase in burst and tonic firing was absent in S100β KO mice. In this experiment, we used conventional S100β KO mice in which compensation for the lack of S100β could have taken place during the development. Therefore, we cannot rule out this possibility. For example, S100A6, which is one of the S100 family and also a Ca^2+^-binding protein, is reported to be expressed and released from astrocytes in the cortex (Cinquina et al., 2025), suggesting that S100A6 might compensate for S100β. However, no study has investigated the expression of S100A6 in S100β KO mice to document such compensation. S100β is reported to be involved in neurological disorders such as Down syndrome (Chen et al., 2014) and epilepsy (Liang et al., 2019), suggesting that it is linked to neuronal regulation. On the other hand, some studies indicated that S100β KO mice do not show major phenotype differences in neuronal development with WT mice (Nishiyama et al., 2002; Bluhm et al., 2015). Moreover, as the comparisons were made between control and pain groups of the same genetic lines (either WT or S100β KO), the observed differences are more likely attributable to the experimental intervention rather than to the global deficiency of S100β itself. However, it should be noted that glial cells involved in the nociceptive pathway also lack S100β in our S100β KO model. Our findings demonstrated a significant increase in glial reactivity in the NVmes in both WT and S100β KO, but no increased activation in the Vc of the KO in the acidic saline-induced pain. Taken together our findings suggest that neuron–glia interactions mediated by S100β in NVmes are responsible for the increased excitability of NVmes neurons and play a predominant role in the development of chronic pain in this model.

Our previous work has shown how astrocytes may modulate the excitability of several types of neurons, including primary afferent somata in NVmes, by altering the level of extracellular Ca^2+^ through the release of S100β (Morquette et al., 2015; Ryczko et al., 2021; Gaudel et al., 2025) and consequently affecting I_NaP_ current. Gaudel et al. (2025) demonstrated that both extracellular BAPTA and S100β increase I_NaP_-dependent SMO amplitude through Na_V_1.6 channels, leading to ectopic discharge. Here, the effects of extracellular application of BAPTA in WT mice were longer and greater in the PAIN groups. Optogenetic stimulation of astrocytes in the NVmes region using GFAP-ChR2-EYFP mice increased slightly (but not significantly) the occurrence of firing in the PAIN group, suggesting that astrocytes in these groups may perhaps be more reactive and release more S100β. I_NaP_ and extracellular Ca^2+^ were not measured in this study, but on the basis of the above-cited studies, we assume that such an increased release could mediate larger effects on NVmes neurons excitability by causing larger decreases of extracellular Ca^2+^ and stronger potentiation of I_NaP_.

Indeed, our findings of increased expression of GFAP in the NVmes of rats and mice strongly support the hypothesis of increased astrocytic reactivity in the acid-induced muscle pain model and are in agreement with many previous reports of astrocytic activation in pain-inducing conditions. NVmes is unique in that it is a centrally located nucleus containing the cell bodies of primary afferents which are normally found in dorsal root ganglia or the trigeminal ganglion. Therefore, its astrocytes are the equivalent of satellite cells in these ganglia. Satellite cells activation has been reported to occur in the trigeminal ganglion in a chronic pain model of burning mouth syndrome (Katagiri et al., 2012, 2023) and astrocytic activation has also been reported in the dorsal spinal horn in association with muscle pain (Borghi et al., 2023) and chronic neuropathic pain (Zhuang et al., 2006). Many studies indicate that inhibition of astrocytes reduces neuropathic pain (Tsuda et al., 2011; Chen et al., 2025; Bao et al., 2026). In the neuropathic pain model rat induced by inferior alveolar nerve ligation, pharmacological suppression of astrocytic activity with sodium fluoroacetate attenuated both the upregulation of GFAP in the Vc, pain-related behaviors and cFos expression in the Vc. These findings suggest that increased GFAP expression is associated with an increase in gliotransmitter-mediated signaling (Okada-Ogawa et al., 2009). However, the gliotransmitter or mechanism mediating their effect has not been formally identified. Here we propose that this effect is achieved through the release of S100β which is in agreement with the observation that reactive gliosis leads to elevated S100β levels (Tanga et al., 2006). This hypothesis is strongly supported by our findings that despite displaying increased astrocytic activation in NVmes like rats and WT mice, there was no increase in excitability of NVmes neurons and expression of cFos in vl-Vi/Vc following acidic saline injections in S100β KO mice. Of significant interest is also the report that S100β KO mice do not develop mechanical hypersensitivity after spinal nerve transection, while on the contrary those overexpressing S100β have a decreased threshold for mechanical responses (Tanga et al., 2006). Thus, S100β may indeed contribute significantly to the development of soreness in jaw muscles as well as in other muscles.

### 4.3 Clinical significance

A characteristic feature of the pain model used in this study is that as little as two injections of acidic saline 2–5 days apart lead to mechanical sensitivity lasting up to 5 weeks (Sluka et al., 2003; Lund et al., 2010; Lee et al., 2025). Recently, the term “nociplastic pain” has been proposed (Trouvin and Perrot, 2019; Tomašević-Todorović and Spasojević, 2023) to refer to this type of chronic pain which does not result from direct tissue damage or nerve injury. Several mechanisms along the neural circuitries involved in signaling pain have been suggested for “chronicisation” of the pain (Garcia-Larrea and Mauguière, 2018; Lin et al., 2022; Katagiri et al., 2023; Ahmed et al., 2025; Sridhar et al., 2026; Wang et al., 2026). Such mechanisms may allow the responsible neural circuitry of pain to engage broader networks over time in association with psychological factors at times (Marando et al., 2025; Zhuo, 2025).

The animal model used in this study does not allow for further exploration of these broader networks, even though acidic saline-induced pain has been associated to an increase in anxiety-like behavior (da Silva et al., 2020). However, previous clinical studies have explored the influence of psychological factors in chronic pain (Manfredini et al., 2010; Celic et al., 2011; Henderson et al., 2016; Hadlandsmyth et al., 2020). This highlights the complexity of the mechanisms and pathways engaging widespread recruitment of brain regions during chronic pain (Yajima et al., 2022; Torres-Rodriguez et al., 2024). Our data demonstrate functional changes in proprioceptive afferents and suggest that pain maintenance could perhaps be prevented if these changes are counteracted rapidly. A potentially interesting approach could be local administration of glutamatergic receptors antagonists or blockers into the muscle to prevent development of chronic pain. These findings may provide new therapeutic options beyond the conventional prescription, such as antidepressants (duloxetine) or anxiolytics (benzodiazepines), often utilized in the clinic (Ferrillo et al., 2022).

Clinically, patients with these types of muscle pain frequently report distress during muscle movement or stretching. This clinical observation supports our finding regarding the involvement of proprioceptors; that is, the excitation of proprioceptors triggered by the stretching of muscle spindles likely evokes the painful sensation. Furthermore, besides the vl-Vi/Vc-related pathway proposed by Sas et al. (2023) based on the antidromic propagation of ectopic firing, the existence of ascending pathways through which ectopic firing in the NVmes propagates orthodromically to higher brain regions can also be postulated. Projections from neurons of the supratrigeminal area which receive a well documented input from MSA in NVmes reach areas, such as the insular cortex in addition to the primary and secondary somatosensory cortices, through several regions of the thalamus, leading to the possibility that neuronal processing of emotion and sensory integration or discrimination is conducted depending on the distinctive thalamic projections (Fujita et al., 2017; Sato et al., 2017; Tsutsumi et al., 2021; Yoshida et al., 2022). Moreover, it is highly plausible that prolonged periods of hyperexcitability in primary afferents may eventually lead to plastic changes in their associated central circuitry apart from the pathway involving the vl-Vi/Vc identified in our current study whether it is within (e.g., from the thalamus to the somatosensory cortex) or outside (e.g., projections to central amygdala or to the insular cortex) of the conventional pain pathway (Lin et al., 2022).

The fact that some languages, including Taiwanese and French, clearly distinguish nociception in muscles from “sngception,” a sensation unique to muscle soreness and describing discomfort (Lin et al., 2018; Hung et al., 2023; Han et al., 2025; Lee et al., 2025) supports the notion that more than one neurological pathway may be employed in chronic muscle pain. If the acidic saline model used in this study truly reflects symptoms of muscle soreness in Temporomandibular disorders (TMD) patients, then this pathway involving the NVmes may be responsible for the unpleasantness often associated with it and potentially accounts for a facet of “sngception,” which is described as pain differentiated from nociceptive pain frameworks.

## 5 Conclusion

This study indicates that acidic saline injection into the masseter muscles induced long-term activation of astrocytes in the NVmes and promoted ectopic firing of NVmes neurons via astrocyte-released S100β. Furthermore, the results also suggested that the vl-Vi/Vc may be involved in the chronic pain induced by acidic saline injection.

## Data Availability

The raw data supporting the conclusions of this article will be made available by the authors, without undue reservation.

## Glossary

ASIC: Acid-sensing ion channels
BAPTA: 1,2-bis(o-aminophenoxy)ethane-N, N, N0, N0-tetraacetic acid tetrasodium salt
[Ca^2+^]_e_: Extracellular Ca^2+^ concentration
cFos-IR: Cellular oncogene fos-immunoreactive
ChR2: Channelrhodopsin 2
CSF: Cerebrospinal fluid
CTL: Control group, injected saline pH 7.4 into the masseter muscles
DOMS: Delayed-onset muscle soreness
EYFP: Enhanced yellow fluorescent protein
GFAP-ChR2-EYFP mice: Mice expressing the channelrhodopsin (ChR2) and enhanced yellow fluorescent protein (EYFP) under the control of the glial fibrillary acidic protein (GFAP) promoter
GFAP-IR: Glial fibrillary acidic protein-immunoreactive
I-V curve: Current–voltage curve
I_NaP_: Sodium persistent current
MSA: Muscle spindle afferents
NVmes: Trigeminal Mesencephalic nucleus
NVmt, Trigeminal motor nucleus
NVsnpr, Trigeminal main sensory nucleus
PAIN: Pain group, injected saline pH 4 into the masseter muscles
PA: Primary afferent
ROI: Region of Interest
PBS: Phosphate-buffered saline
RMP: Resting membrane potential
S100β KO: S100β knockout
SEM: Standard error to the mean
SMO: Subthreshold membrane oscillations
vl-Vi/Vc: Ventrolateral pole of the subnucleus interpolaris/caudalis transition region
WT: Wild type
